# Anterior Segment Analysis and Evaluation of Corneal Biomechanical Properties in Children with Joint Hypermobility

**DOI:** 10.4274/tjo.galenos.2019.28000

**Published:** 2020-04-29

**Authors:** Sadık Etka Bayramoğlu, Nihat Sayın, Dilbade Yıldız Ekinci, Nuray Aktay Ayaz, Mustafa Çakan

**Affiliations:** 1 University of Health Sciences Kanuni Sultan Süleyman Training and Research Hospital, Clinic of Ophthalmology, İstanbul, Turkey; 2 University of Health Sciences Gazi Yaşargil Training and Research Hospital, Clinic of Ophthalmology, Diyarbakır, Turkey; 3 University of Health Sciences Kanuni Sultan Süleyman Training and Research Hospital, Clinic of Pediatric Rheumatology, İstanbul, Turkey; 4 University of Health Sciences Şanlıurfa Training and Research Hospital, Clinic of Pediatric Rheumatology, Şanlıurfa, Turkey

**Keywords:** Cornea, joint hypermobility, ocular response analyzer

## Abstract

**Objectives:**

To compare anterior segment parameters and biomechanical analysis of the cornea in children with joint hypermobility (JH) and healthy children.

**Materials and Methods:**

Cross-sectional case-control study. Fifty eyes of 25 children with JH were compared with 74 eyes of 37 healthy age- and sex-matched controls in terms of refractive, anterior segment topographic, and corneal biomechanical measurements. Axial length (AL) was measured with a Nidek AL-Scan biometry device; corneal-compensated intraocular pressure (IOPcc), Goldmann-correlated IOP (IOPg), corneal hysteresis (CH), and corneal resistance factor (CRF) were measured with a Reichert ocular response analyzer (ORA). Central corneal thickness (CCT), anterior chamber depth (ACD), K1/K2 values, iris diameter, and anterior chamber volume (ACV) were measured with a Sirius topography device.

**Results:**

Mean age in the JH group was 10.56±4.03 years, while that of the control group was 11.27±2.59 years (p=0.23). Spherical equivalent was -0.22±1.02 diopter (D) in the JH group and -0.12±1.12 D in the control group (p=0.60); CCT was 23.01±0.82 µm in the JH group and 23.17±0.82 µm in the control group (p=0.33). There were no significant differences between the two groups in terms of age, sex, IOP, IOPcc, IOPg, CH, CRF, AL, K1, K2, iris diameter, ACD, and ACV.

**Conclusion:**

JH, which causes increased flexibility of the joints, was concluded not to cause a significant change in the corneal biomechanical markers of CRF and CH or in anterior segment topographic parameters.

## Introduction

Hypermobile joints are joints having more flexibility than normal, considering age, gender, and ethnic background. In current rheumatology practice, diagnosis of joint hypermobility (JH) is made by Beighton scoring system, with a score of 4 or more accepted as above normal mobility. This score determines whether there is increased elasticity in 5 body regions: the spine/hip, elbow, fifth metacarpal joint, thumb/wrist, and knee.^1^ Hypermobile joints are prominent features of hereditary connective tissue diseases associated with genetic variations in collagen fibers, such as Marfan Syndrome, hypermobile Ehlers-Danlos syndrome (EDS), formerly known as EDS type 3, osteogenesis imperfecta (OI), and JH syndrome (JHS).^2^ While JH is a simple entity of increased joint motion angles, JHS diagnosis is made by systemic findings in addition to joint and musculoskeletal system symptoms.^1^

JH is more commonly identified in children, and the incidence decreases with age.^3^ In epidemiological studies the prevalence of JH was reported as 5 to 30%.^4,5,6,7,8^ The biomechanical characteristics of the cornea are formed by the stromal layer comprising type I collagen fibers. Descemet’s membrane consists of softer type IV collagen. Therefore, the cornea is one of the target tissues in connective tissue diseases.^9,10,11^

The influence of JH on corneal elasticity and biomechanics has not been clarified. As far as we know, there is no current study investigating the anterior segment parameters of individuals with JH. The aim of our study was to compare the corneal biomechanical and anterior segment parameters of children with JH and healthy children.

## Materials and Methods

In this cross-sectional study, 50 eyes of 25 JH patients admitted to the ophthalmology and pediatric rheumatology clinics of University of Health Sciences Kanuni Sultan Süleyman Training and Research Hospital and 74 eyes of 37 healthy age- and gender-matched controls were compared in terms of refractive, anterior segment topographic, and corneal biomechanical parameters. The study was performed in accordance with the Declaration of Helsinki and with the approval of the ethics committee of University of Health Sciences Kanuni Sultan Süleyman Training and Research Hospital. Written informed consent was obtained from the legal guardians of all children.

All participants underwent ophthalmological examination including refraction, biomicroscopy, and fundus examination. Patients with additional ocular diseases other than refractive error and those with previous intraocular surgery were not included in the study.

Diagnosis of JH was made according to Beighton score of 4 or more (Table 1). A detailed rheumatologic examination of the control group was made and their Beighton score was calculated. All control subjects had scores lower than 4.

Corneal hysteresis (CH), corneal resistance factor (CRF), intraocular pressure (IOP), corneal-compensated IOP (IOPcc), and Goldmann-correlated IOP (IOPg) values were measured using an ocular response analyzer (ORA, Reichert Ophthalmic Instruments, Buffalo, NY, USA). All measurements were taken by an experienced technician. For each eye, 3 measurements with a waveform score above 5 were performed. The mean of these 3 measurements was used in statistical analyses.

Central corneal thickness (CCT), anterior chamber depth (ACD), keratometry values (K1/K2), iris diameter, and anterior chamber volume (ACV) were measured with a Sirius Topography (Sirius®, Costruzione Strumenti Oftalmici, Florence, Italy) device. An experienced technician took 3 measurements for each eye and the measurement with best alignment and fixation was used for statistical analysis.

Axial length (AL) was measured with a Nidek AL-Scan (Nidek, Aichi, Japan) biometry device.

Spherical equivalent (SE) was measured with a Topcon KR-800 Auto-refractometer (Topcon, Tokyo, Japan). IOP was measured with a Nidek NT-510 (Nidek, Aichi, Japan) non-contact tonometer.

Tear film breakup time (TBUT) was recorded in seconds as the time to disintegration of the fluorescein-stained tear film. The mean of 3 separate TBUT measurements was used for statistical analysis. To assess tear production, topical 0.05% proparacaine hydrochloride (Alcaine, Alcon, Puurs, Belgium) was instilled and standard Schirmer test paper was placed under the lateral third of the lower lid. Baseline secretion was measured in mm as the length of paper wetted after 5 min.

Lower eyelid laxity was defined as being able to pull the lower lid more than 10 mm from the globe and delayed return to neutral position. If the inner canthus was above the outer canthus, this finding was assessed as antimongoloid slant.

Statistical analyses were performed using SPSS (SPSS Inc, PASW Statistics for Windows, version 24, Chicago, USA). The normality of distribution of continuous variables was tested by Shapiro-Wilk test. Mann-Whitney U test (for non-normal data) was used for comparison of two independent groups and chi-square test was used to assess relationships between categorical variables. Generalized estimation equation (GEE) analyses were performed to compare groups according to numerical variables while considering the effect of within-subject variations for each eye. P value less than 0.05 was accepted as statistically significant.

## Results

The mean age was 10.6±4.0 years in the JH group and 11.3±2.6 years in the control group (p=0.39). In the JH group, 19 (76.0%) participants were female while in the control group 28 (75.6%) were female (p=0.97). In terms of height and weight, there was no statistically significant difference between the groups (p=0.51, p=0.32). The median values of Beighton score were 5 (range: 4-7) and 2 (range: 0-3) in the JH and control groups, respectively. The difference was significant (p=0.001).

Spherical equivalent was -0.22±1.02 diopters (D) in the JH group and -0.12±1.12 D in the control group (p=0.60), CCT was 549.48±45.88 µm in the JH group and 560.19±35.51 µm in the control group (p=0.118), and axial length was 23.01±0.82 mm in the JH group and 23.17±0.82 mm in the control group (p=0.326). For corneal biomechanical parameters, the JH and control groups had CH values of 11.23±1.89 and 11.52±2.06 (p=0.472) and CRF values of 11.54±1.88 and 12.1±2.07 (p=0.167), respectively. There were no significant differences between the groups in terms of IOP, IOPcc, or IOPg (Table 2). Topographic parameters of the anterior segment such as K1, K2, iris diameter, ACD, and ACV were similar between the JH and control group (Table 3).

The mean TBUT was 9.74±1.48 seconds in the JH group and 9.83±1.46 seconds in the control group (p=0.71). The mean Schirmer test result of the study and control groups were 13.26±2.32 mm and 14.04±2.16 mm, respectively (p=0.06).

Lower eyelid laxity was identified in 4 (16.0%) children in the JH group and 2 (5.4%) children in the control group (p=0.07). Antimongoloid slant was identified in 3 (12.0%) children in the JH group and in 2 (5.4%) children in the control group (p=0.20).

## Discussion

JH may be seen as a part of syndromes like JHS, hypermobile EDS, OI, and Marfan syndrome. JH is not considered a disease but rather a variation of normal. To the best of our knowledge, there is no study evaluating the anterior segment structures of the eyes of children with JH by advanced ocular imaging systems in the PubMed database up to present. In contrast, there are reports concerning pathological ocular findings such as lid laxity, conjunctivochalasis, keratoglobus, keratoconus, lens luxation, pathologic myopia, angioid streaks, scleral thinning, and retinal detachment in these rare syndromes.^12,13,14,15,16^ Also, there are few studies in the literature that perform anterior segment analysis with topography and confocal microscopy imaging methods in patients with JHS, hypermobile EDS, and other EDS types.^13,17,18^ Although JH is a common finding of these rare syndromes, it is not appropriate to compare ocular findings with these syndromes because they are different clinical conditions. Based on our literature search about JH, we found one epidemiological study. In that study ocular findings were identified and questioned by the rheumatologists but imaging with advanced ocular devices was not performed.^19^

In an epidemiological study from Turkey, 861 high school children were evaluated by rheumatologists, and 11.7% of them were found to have JH.^19^ They reported no significant difference for frequency of lid laxity, antimongoloid slant, and myopia between the children with JH and healthy group.^19^ In our study, it was observed that mean SE, frequency of eyelid laxity, and antimongoloid slant were not different between the two groups. We would like to draw attention to the fact that the frequency of myopia was reported according to questioning of the students by a rheumatologist in the study by Seçkin et al.^19^ In other epidemiological studies indicating the incidence of JH in the literature, ocular findings were not included.^3,5,6,7,8^

The main objective of our study was to investigate whether there was a change in the biomechanical and topographic parameters of the cornea in individuals with JH compared to normal individuals. Although there has been only one study investigating the relationship between keratoconus and JH in the literature, keratoconus diagnosis was made according to keratometry and biomicroscopic findings such as central corneal thinning, anterior Fleischer ring, or Vogt lines in that study.^20^ It was reported that the incidence of JH was not increased in patients with keratoconus.^20^ More recent studies showed that biomechanical parameters such as CCT, CH, and CRF and topographic parameters of the cornea were affected in mild keratoconus.^21^ Our study, performed with advanced anterior segment imaging methods, supports the findings of Street et al.^20^ that there were no significant differences between the two groups in terms of biomechanical and topographic parameters and children with JH do not have increased risk of keratoconus.

Although there are studies reporting that the prevalence of JH is high (up to 30%) among healthy children, to our knowledge, there is no other study in the literature examining the anterior segment findings of JH in this age group. We have demonstrated that there was no significant difference between children with JH and healthy controls in terms of anterior segment topography, corneal biomechanical properties, and refractive values using GEE modeling. Our findings suggest that JH is not an ophthalmologically important entity, because anterior segment parameters did not differ from the normal population. Comparative studies with more participants and wider age range would be valuable regarding this topic, which has not been sufficiently researched so far.

## Figures and Tables

**Table 1:**
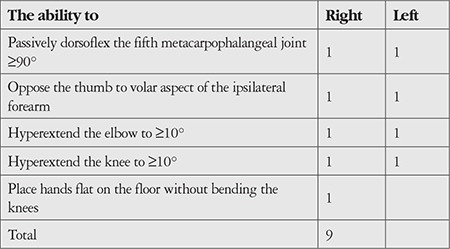
Nine-point Beighton hypermobility score

**Table 2:**
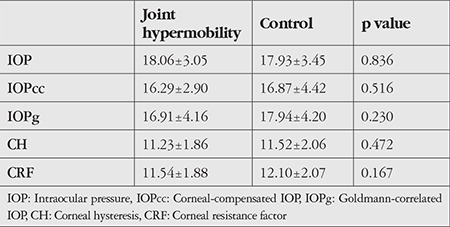
Comparison of intraocular pressure (mmHg) and corneal biomechanical parameters measured with noncontact tonometer and ocular response analyzer

**Table 3:**
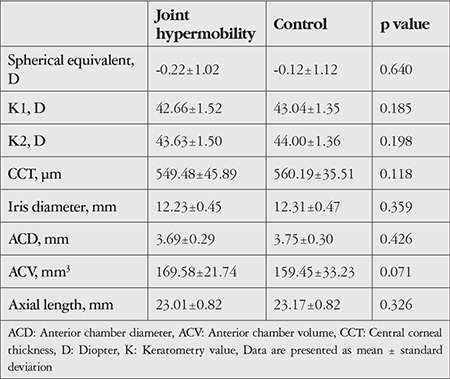
Refractive, topographic, and biometric parameters measured with KR-800 Auto-refractometer, Sirius Topography, and Nidek AL-Scan Biometry devices
